# “We Are Not Being Heard”: Aboriginal Perspectives on Traditional Foods Access and Food Security

**DOI:** 10.1155/2012/130945

**Published:** 2012-12-31

**Authors:** Bethany Elliott, Deepthi Jayatilaka, Contessa Brown, Leslie Varley, Kitty K. Corbett

**Affiliations:** ^1^Population and Public Health, Provincial Health Services Authority, Vancouver, Canada V6Z 2H3; ^2^Heiltsuk Nation, Bella Bella, Canada V0T 1Z0; ^3^Aboriginal Health, Provincial Health Services Authority, Vancouver, Canada V5Z 4C2; ^4^Faculty of Health Science, Simon Fraser University, Burnaby, Canada V5A 1S6

## Abstract

Aboriginal peoples are among the most food insecure groups in Canada, yet their perspectives and knowledge are often sidelined in mainstream food security debates. In order to create food security for all, Aboriginal perspectives must be included in food security research and discourse. This project demonstrates a process in which Aboriginal and non-Aboriginal partners engaged in a culturally appropriate and respectful collaboration, assessing the challenges and barriers to traditional foods access in the urban environment of Vancouver, BC, Canada. The findings highlight local, national, and international actions required to increase access to traditional foods as a means of achieving food security for all people. The paper underscores the interconnectedness of local and global food security issues and highlights challenges as well as solutions with potential to improve food security of both Aboriginal and non-Aboriginal peoples alike.

## 1. Introduction

According to the Food and Agriculture Organization of the United Nations, “Food security exists when all people, at all times, have physical, social, and economic access to sufficient, safe, and nutritious food that meets their dietary needs and food preferences for an active and healthy life” [1]. Although this definition is intended to be inclusive of the dietary needs and food preferences of “all people,” concepts beyond the conventional food system, for example, traditional or country foods, are often overlooked in food security discussions. Two food systems—conventional and traditional—play on food security of indigenous peoples in Canada, referred to as Aboriginal peoples. (The term Aboriginal peoples, as defined by the Canadian Constitution Act, includes Indian—a term which is now commonly replaced by the more accepted term First Nations, Inuit, and Métis peoples). The conventional food system, with which most readers are familiar, prioritizes agribusiness products intended for global consumer markets (market foods). The traditional food system, according to Kuhnlein and Chan [2], “includes all of the food species that are available to a particular culture from local natural resources and the accepted patterns for their use within that culture” and incorporates the sociocultural meanings of these foods, their acquisition, processing, and use within a specific culture. Aboriginal people in Canada have relied on foods from this traditional system since time immemorial, but also access market foods through the conventional system. This unique perspective is often missed in mainstream food security discourse; therefore, we argue that integrating Aboriginal knowledge and perspectives in whole of society strategies is imperative in creating food security for all people.

To date, research on Aboriginal food security has focused on traditional foods within communities living on reserves (tracts of crown land, set apart for the use, and benefit of an Indian band [3]). However, with approximately 60% of the Aboriginal population in Canada now living off reserve [4] a better understanding of the importance of traditional foods in the urban context is crucial to ensuring Aboriginal food security. As a whole, Aboriginal peoples living both on and off reserve are among the most food insecure groups in Canada. In the province where this research project took place (British Columbia, Canada), 41% of Aboriginal households on reservations are food insecure [5]. A Canada-wide survey found that 33% of all off-reserve Aboriginal households are food insecure, compared with only 9% among the general population [6]. 

Both on and off reserves, Aboriginal food insecurity is correlated with many diet-related health concerns. The rate of diabetes among Aboriginal peoples in British Columbia (BC) is 40% higher than the rest of the population, and its incidence continues to rise [7]. The prevalence of heart disease in BC Aboriginal peoples is 25% higher than the general population [7]. In addition, in British Columbia, 54% of Aboriginal people identified themselves as either overweight (31.8%) or obese (22.6%) [7]. Food insecurity and inadequate nutrition play a central role in these chronic health conditions. 

Evidence suggests that traditional foods are more nutritious than conventional diets consisting of market foods. Studies in Canada found that traditional foods contain higher amounts of nutrients and less fat, sodium, and carbohydrates (especially sucrose) than market foods [8–14]. The First Nations Food, Nutrition, and Environment Study examined BC Aboriginal traditional foods and found that dietary quality (i.e., nutrient and energy intake) was improved on days when Aboriginal peoples consumed traditional foods [5]. Due to the health benefits of traditional foods, researchers have warned of the dietary and health risks associated with the “nutrition transition”—a gradual westernization of Aboriginal diets to include more preprocessed market foods and drinks and less traditional foods [2, 5, 11, 15]. However, despite this transition, Aboriginal peoples continue to consume traditional foods [5, 16] and endeavour to pass down traditional knowledge of land and food systems to younger generations, increasing food security, and self-sufficiency within their communities [17].

In light of the importance of traditional foods to health and food security of Aboriginal peoples, and their increasing migration to urban settings, the Aboriginal Health and Food Security programs of the Provincial Health Services Authority (PHSA) undertook a collaborative research project to assess the challenges and solutions to accessing traditional Aboriginal foods in the urban context of Vancouver, BC. The research team had representation from the Aboriginal Health and Food Security programs of PHSA and included a graduate student from the University of Toronto, and a research consultant of Aboriginal heritage. The research team engaged both Aboriginal and non-Aboriginal partners and was guided by a project Advisory Committee consisting primarily of Aboriginal peoples living in Vancouver with knowledge of Aboriginal food security and/or traditional foods. 

This paper describes (1) the collaborative research process, demonstrating how traditional knowledge and “ways of knowing” can be incorporated into food security research; and (2) the findings from this process, highlighting a whole of society approach to enhance access to traditional foods as a means to achieve food security for all peoples. This paper demonstrates the interconnectedness of local and global food security issues and highlights that the challenges and solutions presented have the potential to impact food security of both Aboriginal and non-Aboriginal peoples alike. 

## 2. Methodology

### 2.1. Guiding Principles and Advisory Committee

To ensure that the research project was both meaningful to the Aboriginal community and respectful of their traditional knowledge, the project was guided by the OCAP principles developed by Canada's National Aboriginal Health Organization [18]. The principles are guidelines to help researchers conduct studies that are appropriate and beneficial for Aboriginal peoples and to ensure the ethical application of such research. The OCAP principles are* ownership* of cultural knowledge/data/information by the community or group; the right of Aboriginal peoples to seek *control* of all aspects of research and information management processes that impact them; the right of Aboriginal peoples to *access* information and data about themselves and their communities, and to manage access to their collective information; and the right of Aboriginal peoples to physically *possess *the data collected. 

The OCAP principles are not a prescription for Aboriginal research, but serve as a guide that may be applied and interpreted by the Aboriginal community involved in the research process. The principles were brought to the project Advisory Committee for an open and direct conversation about how these principles should be applied. The Advisory Committee had no reservations about sharing their knowledge, preferring to build the research relationship on trust and communication rather than rules and regulations dictating knowledge transfer. Although the Advisory Committee interpreted the OCAP principles more loosely than some communities, this conversation was essential to ensure a common understanding and to reiterate the commitment to a respectful research process. 

To ensure that the project was relevant, respectful, and important to urban Aboriginal peoples, the Advisory Committee was involved in every stage of the project—from developing the research question and methodology to the analysis, conclusions, and dissemination. The Advisory Committee met in person approximately once a month and offered feedback between meetings via telephone or email. At monthly meetings, the group always shared a healthy meal together, and whenever possible, traditional foods were served. Advisory Committee members were acknowledged with honoraria for their knowledge and time.

### 2.2. Sample and Recruitment

Intergenerational teaching/learning was an identified priority for the Advisory Committee and their associated community groups/organizations. To facilitate such learning, the project aimed to recruit 12 Aboriginal young people (youth) aged 19–30 years old and six community-identified Elders (or “Elders-in-Training” to participate in the study. (Elders-in-Training were Aboriginal peoples who were too young to be recognized as Elders, but were knowledgeable in traditional practices and active teachers in their communities.) 

Two Advisory Committee members were particularly active in youth recruitment, registering interested participants through Aboriginal youth programs in Vancouver and presentations at community gatherings. The number of youth interested in participating exceeded study capacity; therefore, 15 youth were randomly selected to participate. The research team intentionally oversampled, anticipating that some youth may not attend. Youth who were not selected to participate were invited to stay involved by receiving project updates and final reports. For their participation, youth received a monetary honorarium consistent with other research projects and in keeping with the OCAP principles.

Through their personal and professional networks, the Advisory Committee identified respected Elders who were knowledgeable in traditional foods and practices. All six Elders contacted agreed to participate in the study and received an honorarium for their contribution of knowledge. 

In total, fifteen youth and six Elders provided informed consent and participated in the study. 

### 2.3. Research Design and Knowledge Generation

A common critique of traditional foods studies is the priority of western science over traditional knowledge and Aboriginal perspectives [19–23]. To avoid this pitfall, the research team and Advisory Committee prioritized culturally relevant practices and aimed to generate knowledge based on the lived experiences of urban Aboriginal peoples. A participatory research study drawing on narrative inquiry approaches was designed, and received ethics approval from the University of Toronto. Narrative inquiry is a qualitative research approach that incorporates participants' lived experiences through stories. Knowledge is co-constructed as participants tell their stories and the researcher listens, asks questions, and interprets what he/she hears [24]. In this study, narratives were employed to generate knowledge based in urban Aboriginal experiences. The narratives were created using the Story/Dialogue Method of gathering experiential knowledge developed by Labonte and Feather [25]. This method was selected due to its synchrony with the traditions, practices, and ways of knowing in many Aboriginal cultures. 

The Story/Dialogue Method documents experiential knowledge through sharing of stories in a structured format. In small “Story Groups,” participants are asked to share a story from their personal experience. The group then move through a series of questions, progressively reaching deeper levels of analysis of the story. Four categories of questions are discussed:

1. *“What?”* questions clarify the details of the story;
2. *“Why?”* questions seek an explanation for the events of the story;
3. *“So What?”* questions synthesize what was learned from the story; and
4. *“Now What?”* questions cause participants to consider future actions to improve the outcome of similar stories.

After participants move through all four levels of questions, they collectively create “Insight Cards” to capture the key discussion points for each set of questions. Each insight is written on a separate card, and participants work together to arrange the cards into categories or themes. The Story Groups then create a summary statement (a “Theory Note”) for each grouping, followed by a comprehensive summary statement linking all the themes together (a “Composite Theory Note”). 

The Advisory Committee reviewed the Story/Dialogue Method and appreciated its resonance with storytelling—a deeply rooted cultural practice. However, the method was deemed too structured for an Aboriginal audience. Therefore, the method was adapted so participants were not responsible for the structured questioning and recording of comments and insights. Instead, trained facilitators were incorporated to gently guide the discussion to deeper levels of analysis. The facilitators also took over the majority of the note taking tasks, allowing participants to fully engage in the conversation. Although participants still created Insight Cards and organized the cards into categories, the term “Theory Note” was not used with participants, as it was deemed overly academic and potentially off-putting for a community setting. Due to time limitations of the day, the final summary (“Composite Theory Note”) was omitted. Two non-Aboriginal and four Aboriginal peoples were recruited and trained in the adapted storytelling methodology.

The adapted Story/Dialogue Method proved to be effective in reaching the research aims of the project and incorporating traditional ways of knowing and generating knowledge. Other cultural traditions were also incorporated into the methodology—traditional foods were provided for lunch; Elders offered an opening welcome and closing prayer song; and talking circles were used to share introductions, personal stories, and feedback. The storytelling was held at the Aboriginal Friendship Centre in Vancouver, a well-known hub for Aboriginal peoples in the city. 

### 2.4. Analysis

A major strength of the chosen methodology was the ability for participants to conduct the primary analysis of the themes emerging from their own stories and experiences. Participants collectively identified the key insights that they determined to be important to share with a wider audience. Each Story Group created Insight Cards. Some groups compiled their insight cards into one written summary, while other groups shared their summary orally. In an effort to incorporate culturally relevant approaches, facilitators readily accepted oral sharing of knowledge and took notes on what was shared. 

Following the storytelling day, the research team conducted a secondary analysis by compiling the Insight Cards and written and oral summaries from all Story Groups into one cohesive story and three variations of a visual model. Participant quotes were taken directly from facilitator's notes and the Insight Cards developed by the story groups. The story and visual models were reviewed by the Advisory Committee to ensure that they were both accurate and culturally appropriate. After incorporating Advisory Committee feedback and selecting the most appropriate model, the Committee and all participants were invited to a follow-up event held one month after the initial storytelling day. Participants offered feedback on the accuracy and clarity of the messages presented. This participatory approach to analysis allowed participants to “speak for themselves” and control how their stories and ideas were represented to a wider audience. 

## 3. Results

**Figure 1:**
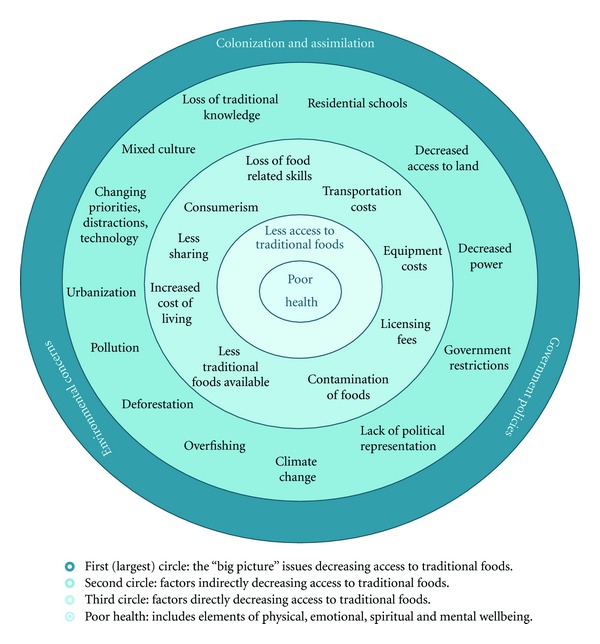
Factors that limit access to traditional foods in the city.

**Figure 2:**
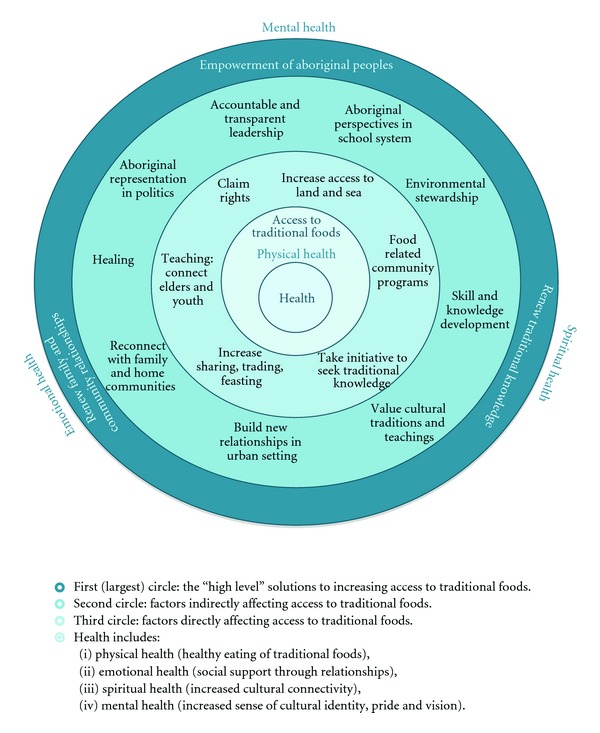
Actions to increase access to traditional foods in the city.

Participant-identified challenges and solutions to accessing traditional foods in Vancouver are presented in Figures 1 and 2. As highlighted in the reviewed literature and echoed by our participants, access to traditional foods improves health. As such, both diagrams are centred on the health of Aboriginal peoples. Participants noted that the Aboriginal concept of health is holistic and includes physical, emotional, mental, and spiritual health—leading to overall wellbeing. Traditional foods, due to their connectivity with cultural practices and traditional knowledge, impact not only physical health, but also emotional, mental, and spiritual health. 

### 3.1. Factors That Limit Access to Traditional Foods in the City

Just as consumption of traditional foods improves the diet and health of Aboriginal peoples, less access to traditional foods leads to a poorer diet and poorer health. The many factors that impede access to traditional foods are organized in concentric circles around “poor health” in Figure 1, with more direct influences placed closer to the centre. The circular representation, rather than a linear model, was purposely selected by the Advisory Committee because of the interrelated and overlapping nature of these factors over time.

Living in an urban centre brings unique challenges to accessing traditional foods. Participants stated that traditional foods are often shared through family networks, but due to distance or disconnection from their family and/or home community, they receive less traditional foods when they live in the city. Some participants suggested returning to the reserve “to learn and get traditional teachings,” while one youth participant noted that her family chose to distance themselves from the community: “Mom did not like the negative things on the reserve, so that is why she kept me away from the reserve.” Participants also noted that sharing of traditional foods has been affected by the increase in consumer mentality in the city, stating “Previously, it was very much about sharing. Now, everything is about money… We are measured only by how much we can buy.” Youth raised in urban environments stated that they grew up in a “mixed culture” and struggled to maintain traditional ways of living, while attempting to succeed in a fast-paced urban environment: “We need to learn how to live our old ways and new ways together, and still be successful as Native Peoples.” Youth said that in the city they have fewer opportunities to learn from Elders, and many stated they did not have the knowledge or skills to gather and prepare traditional foods.

While traditional knowledge continues to be preserved and shared in Aboriginal communities, participants stated that this knowledge is being lost over generations. Participants traced this gradual loss of traditional knowledge and skills back to residential schools, where, for generations, children were removed from their families and communities and forced to learn European ways of living, eating, and speaking. As one Elder shared, “It all comes back to residential schools, but we have the knowledge in our DNA. It's our duty to share it before it is extinct.” As highlighted in the literature, assimilation practices initiated a “nutrition transition” among Aboriginal peoples by introducing them to less healthy non-traditional foods. One youth described the nutrition transition as the introduction to the “five white sins: flour, salt, sugar, alcohol, and lard.” Participants underscored that colonization and forced assimilation have had a lasting impact on traditional knowledge and family dynamics, both of which impact access to traditional foods. 

At a macrolevel, participants identified that government policies and the changing environment continue to impact access to traditional foods. For example, many participants stressed that “the Department of Fisheries and Oceans does not allow us to fish off our territory”—pointing to the reservation boundaries that contain Aboriginal peoples and limit access to fishing, hunting, and gathering grounds, particularly for those living off reserve. Discussions on government policies also highlighted licensing fees that make hunting and fishing too expensive for many Aboriginal peoples, impacting their ability to afford and share traditional foods: “Now we need licenses to collect everything from oysters to crab to kytons. It wasn't like this before. We could just go and collect what we wanted.” Furthermore, continuing increases in transportation and equipment costs leave many families unable to afford the equipment or gas needed to hunt, fish, or gather foods. As one Elder stated, “Traditional foods should not be a privilege (for the wealthy), it should be available for all!” Lack of Aboriginal control over how land and waterways are being used, environmental pollutants that contaminate food and food sources, deforestation, climate change, and overfishing were all discussed as factors reducing the availability of traditional foods. Participants felt that not enough people were raising these issues with decision makers and noted the insufficient political representation of Aboriginal peoples in the Canadian government as a significant issue. 

Overall, the challenges to accessing traditional foods discussed through storytelling match those identified in the literature reviewed, despite the fact that most of the previous Canadian studies focused on reserve or northern communities. However, participants highlighted the additional challenge of living in an urban setting. Costs of living are higher in the city; there is less opportunity for traditional teachings, and youth in particular stated that it is difficult to balance urban living with traditional values. 

### 3.2. Solutions to Increase Access to Traditional Foods in the City

The solutions to increasing access to traditional foods are represented in Figure 2. Similar to Figure 1, health is at the centre. The more indirect, overarching solutions identified by participants—empowerment of Aboriginal peoples, renewal of traditional knowledge, and renewal of family and community relationships—are located in the outermost circle. These overarching solutions not only impact traditional foods access and physical health (nutrition), but also have positive effects on mental, emotional, and spiritual health. Participants emphasized this holistic view of health.

Overall, participants stressed the need to collectively heal from the past injustices of colonization and assimilation. As one participant highlighted, “We must learn from the past and forgive in order to be the best we can be.” Renewal of family and community relationships was seen as important for connecting youth and Elders, facilitating the teaching of traditional knowledge to the younger generation, encouraging the sharing and trade of traditional foods, and celebrating together in cultural feasts. According to participants, “feasts are a fun and engaging way to bring family and community together” and they “provide an opportunity for young people to serve, associate with, and show respect for Elders.” When urban Aboriginal peoples are far from their family or home community, participants stressed the importance of “building relationships with the surrounding Aboriginal communities and peoples who are native to the area” to facilitate teaching, sharing, and trading. 

Elders underlined the importance of youth valuing traditional knowledge and investing time and energy into learning and gaining skills. As one Elder stressed: “Be there to experience and be shown. Physically do it!” Similarly, youth acknowledged the need to take initiative in seeking traditional knowledge from Aboriginal community programs/events, relatives, and Elders. As one Story Group expressed, youth should “communicate with Elders about traditional food, rather than expecting them to come to you.” However, they recognized that this is not always easy: “Elders are role models and want to pass on their knowledge, but some youth do not have the opportunities or resources to learn from them.” Participants also noted that traditional knowledge and practices are closely linked to environmental stewardship, stating that it could help protecting food sources from mismanagement, over-extraction, and pollution.

Participants saw the renewal of traditional knowledge as key to Aboriginal empowerment. Some Elders present had gone to residential schools, and through that system, “language, culture, and pride in traditions were lost.” Youth wanted to see an appreciation of indigenous knowledge in both Aboriginal and non-Aboriginal communities, and an inclusion of Aboriginal perspectives, history, and culture in the school curriculum. For example, some youth said they had switched from the “regular” social studies class to the First Nations option and stressed the importance of Aboriginal and non-Aboriginal students learning “both sides of the story” in Canadian history.

Elders and youth emphasized the need for accountable and transparent leadership across the board, empowering Aboriginal peoples to claim their rights and access land and water systems. The need for Aboriginal voice in public policy making was highlighted, with participants firmly stating, “We are not being heard!” They saw political representation as vital to improving access to traditional foods—not only in urban settings, but for all Aboriginal peoples. 

Solutions for increasing access to traditional foods are less common in academic literature. However, participants in the storytelling event were more excited by discussing potential solutions and actions than by reiterating challenges. By allowing participants to direct the conversation, the discussion naturally shifted to focus on positive actions that could be taken at local, regional, and national levels. 

### 3.3. Summary of Findings

**Figure 3:**
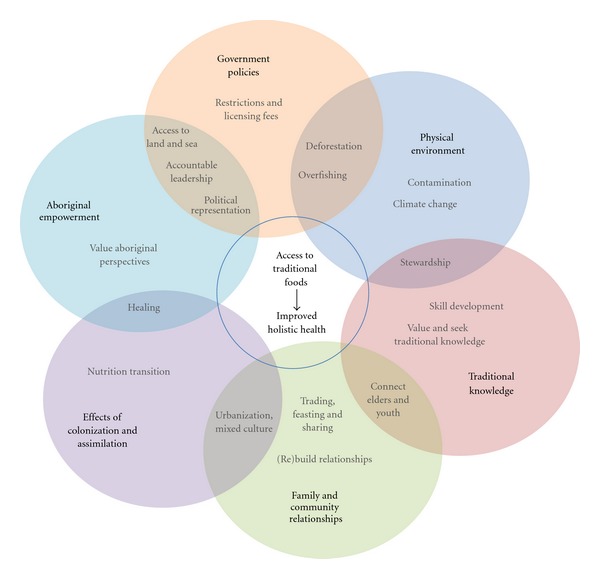
Summary of main factors that impact access to traditional foods in vancouver.

Figure 3 summarizes the main factors that impact access to traditional foods in Vancouver, as described by study participants. Challenges and solutions are brought together to demonstrate how all factors are interrelated and collectively impact access to traditional foods and holistic health. Participants identified a comprehensive range of influences, from local (e.g., interpersonal relationships), to regional (e.g., government policies), to global (e.g., environmental concerns). 

Many of the challenges and solutions to accessing traditional foods articulated in the storytelling workshop parallel challenges and solutions of food security more broadly. The influence of societal priorities and values, social support, environmental concerns, and government policy impact the food security of all people. Community and family connections play an important role in community and household food security among both Aboriginal and non-Aboriginal peoples, and traditional knowledge of Aboriginal groups offers valuable insight into the changes occurring in our environment and food sources. The comprehensive range of factors identified by the research participants can be applied beyond the Aboriginal context and provide insights for food security in a diversity of settings.

## 4. Conclusions

The value placed on traditional knowledge, taking time to build respectful relationships between Aboriginal and non-Aboriginal partners, and the incorporation of Aboriginal perspectives in the research design, implementation, and analysis were imperatives in the success of the project. The trusting and open relationship between Aboriginal and non-Aboriginal partners allowed for the incorporation of traditional knowledge and Aboriginal perspectives in all aspects of the research process. Incorporating traditional practices—such as gift giving, sharing circles, and storytelling—not only helped to strengthen relationships, but also strengthened the research findings. The adapted Story/Dialogue Method resonated with the cultures and traditions of research participants and enabled participants to determine the content of discussion and final reports. Where other research projects focused on challenges to accessing traditional foods, participants in the storytelling event preferred to talk about—and even plan to implement—solutions to increasing access in the city. The adapted Story/Dialogue Method not only gave participants ownership over the research content, but also fostered a renewed interest and excitement about traditional foods, resulting in grassroots actions by the participants. Many participants resolved to seek out traditional food and knowledge. One youth collected the contact information of an Elder in order to connect with her for teaching and learning opportunities. Collectively, the group started a Facebook page to promote local trading of traditional foods, brainstormed a television series to pitch to Aboriginal Peoples Television Network, and promoted local community gardens that provide traditional teachings. However, participants also recognized that many of the challenges and solutions discussed are beyond their control. 

The knowledge generated from the project highlights the need for a whole of society response to increase and sustain access to traditional foods as a means to achieve Aboriginal food security. Participants highlighted a wide range of factors affecting their access to traditional food, from local to international influences. They clearly articulated that some factors lie within their immediate communities, such as family and community relationships, teaching and learning traditional knowledge, and strong local leadership. Other factors rest with the national and international community, such as government policies and regulations that impact traditional food sources, the physical environment, and impacts of climate change and contamination, and the “nutrition transition” from traditional to conventional diets. To fully appreciate the factors impacting access to traditional foods, it is important to look beyond the individual or their communities and incorporate the regional, national, and international factors at play. 

Including Aboriginal knowledge and perspectives in food security research and discourse not only identifies unique challenges and solutions, but also highlights issues that are of relevance to all of society. In every society, food security is impacted by local factors such as social relationships and food skills and knowledge, and broader factors such as government policies, the physical environment, dependence on the conventional food system, and adequate voice and political representation of marginalized or vulnerable (i.e., food insecure) populations. The participants comprehensively articulated the interconnectedness of local and global factors impacting not only access to traditional foods, but also the food security of Aboriginal and non-Aboriginal peoples alike. In order to holistically address food security issues and achieve food security for all, Aboriginal peoples must be engaged as equal partners, with their knowledge and worldview prevailing in decisions within their own communities and also being solicited and incorporated into society-wide and global policies.
